# Reviewed article: Silva FO, Carvalho AL, Schnepper GGM, Marchesi LD, Leite IR, Endo LV, Lima CDVS, Massa KHC. Association between diagnosis of depression and health servisse coverage: a multilevel analysis of the National Health Survey, Brazil, 2019. Epidemiol Serv Saude. 2026:35;e20250666

**DOI:** 10.1590/S2237-96222026v35e20250666.a

**Published:** 2026-02-27

**Authors:** Osmar Cardoso

**Affiliations:** 1 Universidade Federal do Piauí, Teresina, PI, Brazil

## Peer review A

## Questionnaire

Does the manuscript contain new and significant information to justify publication? Yes

Does the Abstract (Summary) clearly and accurately describe the content of the article? Yes

Is the problem significant and concisely stated? Yes

Are the methods described comprehensively? Yes

Are the interpretations and conclusions justified by the results? Yes

Is adequate reference made to other work in the field? Yes

## Manuscript Structure

Length of article is: Adequate

Number of tables is: Adequate

Number of figures is: Adequate

Please state any conflict(s) of interest that you have in relation to the review of this paper. None

Interest: Excellent

Quality: Excellent

Originality: Excellent

Overall: Excellent

Recommendation: Minor Revision

## Comments

Considero o trabalho muito pertinente e adequado às condições da regionalização. Tenho algumas preocupações e sugestões para o aprimoramento e a apresentação de resultados que podem aproveitar mais esses dados, sugerindo mais focalização nas causas que causam essa diferença nas coberturas e diagnósticos e cuidados da depressão na população.

Minhas preocupações com o artigo são:

1 - As tabelas devem ser mais autoexplicativas, na legenda, pq está muito simplificado.

2 - Deveria haver mais propostas de associações entre as variáveis, como por exemplo, os brancos tem mais depressão, mas será que não é pq eles moram em locais com mais coberturas dos serviços de saúde?

3 - Poderia ser apresentado sugestões sobre os locais dos vazios assistenciais e as sugestões de cobertura

